# The protective effect of grit on clinical nurses’ occupational psychological distress: Mediating and suppressing effects of Hope

**DOI:** 10.3389/fpsyg.2022.1019655

**Published:** 2022-09-29

**Authors:** Xueping Peng, Dongmei Wu

**Affiliations:** ^1^School of Nursing, Chengdu Medical College, Chengdu, China; ^2^MOE Key Laboratory for Neuroinformation, Department of Nursing, The Clinical Hospital of Chengdu Brain Science Institute, University of Electronic Science and Technology of China, Chengdu, China

**Keywords:** grit, hope, depression, anxiety, stress, clinical nurse, Chinese

## Abstract

As at a high-risk group of psychological distress, nurses generally experience varying degrees of stress, anxiety, and depression. This paper identifies the positive factors that may negatively regulate the psychological pain of clinical nurses and their mechanisms of action, providing reliable references for clinical nurse support management. The effects and mechanisms of hope and the two components of grit consistency of interest and perseverance of effort) on clinical nurses’ psychological distress (depression, anxiety, and stress) were observed in this study. A total of 635 Chinese clinical nurses (90.4% female) completed an anonymous questionnaire for the survey. As expected, hope, consistency of interest, and perseverance of effort were negatively correlated with the three indicators of psychological distress (*r* = −0.21 ~ −0.38, *p* < 0.01). Path analysis results showed that hope significantly mediated the negative effect of consistency of interest on psychological distress, with an effect of 12.96%. Hope also covered up the perseverance of effort on psychological distress, the effect of 110.63%. In the influence of consistency of interest and perseverance of effort on psychological distress, hope contributed a vital mediating. Based on these results, it can be concluded that grit and hope have protective effects on psychological distress in clinical nurses. Significantly increasing the level of hope or grit may effectively prevent and reduce psychological distress in clinical nurses.

## Introduction

Clinical nursing is a very challenging and high-risk job. Clinical nurses not only directly face the illness or death of patients but also face all kinds of suffering of patients and their family caregivers in their daily work. This highly stressful and emotional work can increase physical and emotional stress for nurses (Byers, 2017). Meanwhile, the changes and challenges to health care brought about by the dramatic increase in natural and artificial disasters also place an additional psychosocial burden on nurses (Deng et al., 2019; Hill et al., 2022). Many studies have assessed and reported high levels of stress (Kemper et al., 2011; McTiernan and McDonald, 2015; Mahdizadeh et al., 2016), depressive symptoms (Letvak et al., 2012; Cheung and Yip, 2015) and anxiety symptoms (Gao et al., 2012; Alhroub et al., 2022) among health care workers worldwide. Despite cultural and organizational differences, nurses worldwide are a population with higher stress and emotional symptoms (Creedy et al., 2017; Poursadeghiyan et al., 2017; Han et al., 2020; Abraham et al., 2021; Jokwiro et al., 2021; Marvaldi et al., 2021). Stress, anxiety, and depression are collectively called psychological distress. These psychological disturbances may be related to nurses’ job performance (Ford et al., 2011; Ivandic et al., 2017), job satisfaction (Zangaro and Soeken, 2007; Lu et al., 2019), job burnout (Glasberg et al., 2007; Braithwaite, 2008; Koutsimani et al., 2019; Stelnicki et al., 2021), turnover intention, and subsequent abandonment of employment (Aiken et al., 2002; Kim and Kim, 2011; Fried et al., 2013). Therefore, it is a good choice for the future hospital occupational health management reform to strengthen the intervention of psychological distress of nursing staff. Clinical nurses’ intervention measures for psychological distress are mainly social psychological interventions, such as social support, work-environment improvement, education, and coping interventions (Petzold et al., 2020; Hickey, 2022). Of these, coping interventions are considered the most preferred (Colville et al., 2017) and are dominated by mindfulness-based interventions (Ghawadra et al., 2019; Ramachandran et al., 2022). Furthermore, many nurses can continue to provide high-quality care and have satisfactory careers despite the multiple challenges of the current medical environment, which is consequenced by the diverse responses of individuals to stressors (Mark and Smith, 2012). As a result, great interest is in the positive traits and psychological resources that may help nurses cope with the challenges in work, such as self-efficacy, hope, optimism, resilience, and grit (Young and Rushton, 2017; Zhou et al., 2017; Meyer et al., 2020; Huang et al., 2021), increasing those resources may help reduce clinical nurses’ vulnerability to psychological distress (Hjemdal et al., 2006; Abraham et al., 2021). Among these, grit and hope may have such effects and mechanisms.

### The negative relationship between grit and psychological distress

Grit is defined in positive psychology as “passion and perseverance for long-term goals” (Duckworth et al., 2007). As a psychological trait that has a positive effect on mental health, grit has been widely discussed in academic circles in recent years and has been conceptualized as a positive psychological trait related to motivation and perseverance (Hill et al., 2016; Sharkey et al., 2018). Structurally, grit consists of two dimensions: “consistency of interest in long-term goals and perseverance of effort in pursuing these long-term goals” (Duckworth et al., 2007; Duckworth and Quinn, 2009). Evidence suggests that people with more grit show greater motivation and endurance (Hill et al., 2016). From the perspective of resource theory, grit is an essential positive psychological resource for individuals to cope with stress. Individuals with higher levels of grit exhibit more positive psychological traits and behaviors, which may also reduce the probability of psychological distress (Liu et al., 2022). The negative relationship between grit and stress, anxiety, and depression has been demonstrated (Singh and Jha, 2008). In the Datu (2021) review, grit was associated with lower levels of depression and stress in individuals and lower anxiety sensitivity and anxiety levels.

However, some studies have found that the two dimensions of grit (consistency of interest and perseverance of effort) may not all have the same effect on mental distress. Zhong et al. (2018) found that grit’s consistency of interest (CI) and perseverance of effort (PE) subscales correlate differently with mental health. This founding is consistent with the report by Masuyama et al. (2021) that the CI subscale significantly negatively predicts mental health outcomes, whereas PE had no significant effect. It seems that CI is more strongly associated with mental health than PE. In any case, grit is considered an essential quality of nurses (Tyer-Viola, 2019). However, there is still a lack of understanding as to whether grit negatively regulates psychological distress in clinical nurses and whether other psychological resources, such as hope, influence the effect of grit on psychological distress. Further research is needed.

### The negative relationship between hope and psychological distress

Snyder and Forsyth (1991) defined hope as “a positive motivational state.” DuFault and Martocchio (1985) described hope as “a multi-dimensional dynamic life force characterized by a confident yet uncertain expectation of achieving good.” Structurally, hope is defined as an individual’s perception of the identification of the paths to achieve their goals (pathways thinking) and the ability to exploit these paths (agency thinking; Snyder, 2002). Hope is also to be conceptualized as an intrinsic force associated with better emotional and stress coping (Lu and Cui, 2016). It not only plays an indispensable role in developing positive anticipatory goals but is also an essential resource to help individuals cope with physical and mental pain (Olsman, 2020). Berendes et al. (2010) found that hope can reduce stress and depression in individuals; Alacorn et al. (2013) combined results from 26 studies involving 4,023 participants and found a significant negative association between hope and depression. These results proved that hope is a protective factor for mental health and that individuals with more hope are more likely to adopt positive attitudes and strategies to face difficulties and challenges and are less likely to have negative emotions (Kalliath and Morris, 2002; Duggleby et al., 2009; Coppock et al., 2010). Hope may also be an essential resource for clinical nurses confronting work challenges and self-regulation. However, few studies have focused on nurses’ level of hope and the relationship between nurses’ station of hope and their psychological distress. As researchers increasingly recognize the value of clinical nurses’ mental health, exploring hope’s possible, supporting effect on their psychological distress is vital.

### The relationship between grit and hope

Grit and hope are both positive psychological factors for the future (Snyder, 2002; Duckworth et al., 2007), and studies have reported a positive correlation between them (Vela et al., 2018; Clement et al., 2020; Lee et al., 2022). Hodge et al. (2019) found that hope moderates the effect of grit on mental health in disadvantaged groups, with individuals with higher levels of grit having greater hope and a lower risk of psychological distress. Grit and hope may have a common mechanism in protecting against psychological pain. Based on the relationship between grit and hope and their uniform effects on psychological distress, hope, as a coping resource, may improve mental state through a goal-oriented motivational process and further enhance the protective effect of grit on psychological distress.

### The culturability of grit and hope

Duckworth and colleagues (Duckworth et al., 2007; Duckworth and Gross, 2014; Duckworth, 2016) have repeatedly emphasized that grit is a positive personal trait that can be cultivated. But current interventions that promote grit still occur, mainly in academic settings (Eskreis-Winkler, 2015; Keegan, 2017; Tang et al., 2019). Researchers have discussed cognitive behavioral therapy and value-based interventions such as Acceptance and Commitment Therapy. These methods may improve grit by helping individuals improve their thoughts and behaviors consistent with their long-term goals (Eskreis-Winkler et al., 2016; Sharkey et al., 2018). As practical interventions do not exist currently, grit is considered likely to be more appropriate for talent screening assessment (Sharkey et al., 2017).

According to Luthans and Broad (2022) psychological capital theory, hope is a positive mental resource that can be easily measured, nurtured, and effectively managed. Damreihani et al. (2018) demonstrated that 1 month of positive psychological intervention could substantially increase the level of hope of mothers of children with cancer. More importantly, the hope intervention can raise the level of hope, and at the same time, it can improve mental health outcomes accompany (Khodabakhshi et al., 2017; KhalediSardashti et al., 2018). In a study by Fallah et al. (2011), eight spiritually based group education and psychological interventions improved the hope and mental health of women with breast cancer. Hope-based interventions by Cheavens et al. (2006) increased hope in life and reduced anxiety and depression in a community sample. Rashidipour-Fard et al. (2017), who performed hope therapy on 50 hypertensive patients, found that the scores of anxiety and depression in the hope therapy group were significantly lower than those in the control group before and after the intervention. The hoped-for intervention seems to offer a more viable approach to human resources management and deserves attention.

### Present study

**Figure 1:**
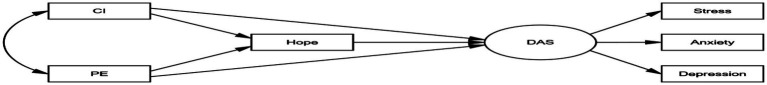
Hypothetical model.

Grit and hope are favorable traits and psychological resources and can be cultivated. But we know little about their effects and mechanisms on clinical nurses’ psychological distress. This study aims to enhance the understanding of grit and hope’s role in the psychological distress of clinical nurses and to provide crucial practical help for clinical nurses’ mental health support. The evidence obtained in the study may also help develop psychological health-related strategies and policies. Based on the existing research and theory, we tried to put forward three specific hypotheses: grit and clinical nurse psychological distress is a significantly negative correlation. Hope is significantly negatively correlated with the psychological distress of clinical nurses. Hope mediates the relationship between two components of grit (CI and PE) and psychological distress (depression-anxiety-stress) in clinical nurses. We also constructed a mediation model (Figure 1) to test the mediating role of hope between CI, PE, and depression-anxiety-stress (DAS).

## Materials and methods

### Participants

Since the research data collection was conducted at a specific time, this study was a cross-sectional survey. 682 clinical nurses, the study group members, were recruited from different hospitals in Western China and met the criteria through online questionnaires. All participants signed informed consent and volunteered to participate in this study. The inclusion criteria were: (a) obtained the professional qualification certificate of clinical nurses of the People’s Republic of China; (b) currently engaged in clinical nursing or clinical nursing management; (c) have no previous or current diagnosis of mental symptoms/diseases and no diagnosis of drug or alcohol dependence; (d) have basic telephone or computer skills; (e) volunteered to participate in the study. Study participants who failed to complete the survey were excluded. Of the 682 questionnaires collected, 635 were valid (47 nurses failed to complete the questionnaire), and the response rate was 93.11%. Table 1 lists the characteristics of the participants.

**Table 1:** Demographic characteristics of research objects (*n* = 635).

Variable	Sample	Grit scores			Hope scores			n (%)	Mean (SD)	t/F	*p*	Mean (SD)	t/F	*p*
Gender			*t* = 1.898	0.058		*t* = 1.414	0.158
Male	61 (9.6)	28.1 (4.31)			22.48 (2.94)		
Female	574 (90.4)	27.03 (4.15)			21.81 (3.54)		
Age (years)			*t* = 0.737	0.461		*t* = 3.246	0.001
<30	289 (45.5)	27 (4.28)			21.39 (3.43)		
≥30	346 (54.5)	27.25 (4.08)			22.28 (3.49)		
Length of nursing work (years)			*t* = 0.380	0.704		*t* = 2.693	0.007
<10	367 (57.8)	27.19 (4.17)			21.56 (3.32)		
≥10	268 (42.2)	27.06 (4.18)			22.31 (3.68)		
Education			*t* = 0.79 7	0.426		*t* = 0.689	0.492
Below bachelor degree	274 (43.1)	27.29 (3.98)			21.77 (3.55)		
Bachelor degree or above	361 (56.9)	27.02 (4.31)			21.96 (3.44)		
Positional ranks			*F* = 1.543	0.215		*F* = 5.279	0.005
Nurse	145 (22.8)	27.06 (3.99)			21.2 (3.47)		
Nurse practitioner	333 (52.4)	26.94 (4.28)			21.88 (3.48)		
Nurse-in-charge and above	157 (24.8)	27.64 (4.08)			22.5 (3.44)		
Hospital type			*t* = 2.693	0.007		*t* = 0.345	0.73
general hospital	304 (47.9)	26.67 (4.09)			21.83 (3.74)		
Psychiatric hospital	331 (52.1)	27.56 (4.2)			21.92 (3.25)		
Working department 1			*F* = 3.785	0.01		*F* = 5.139	0.002
Working department 2			*F* = 2.107	0.123		*F* = 6.776	0.001
internal medicine	113 (17.8)	26.06 (3.83)			20.82 (3.87)		
Surgery	130 (20.5)	27.12 (4.11)			22.48 (3.45)		
Psychiatry Department	331 (52.1)	27.56 (4.2)			21.92 (3.25)		
Others	60 (9.6)	26.87 (4.42)			22.28 (3.74)		

Working Department 1, which includes all departments for analysis.

Working Department 2, which includes all departments except psychiatry for analysis.

### Measures

The study used a convenient sampling method to investigate the clinical nurses in six general and psychiatric hospitals in Western China. All the tests were conducted in Mandarin Chinese.

### Demographic variables

The self-designed demographic questionnaire was utilized in this study to collect the characteristics of participants, including gender (male, female), age, educational background (college degree, bachelor’s degree, or graduate degree), length of nursing service, hospital type, working department, and positional ranks were collected.

### Depression-anxiety-stress scale (DASS)

The scale was initially developed by Lovibond and Lovibond (1995), simplified by Antony et al. (1998), and sinicized and validated by Gong et al. (2010). DASS-21 consisted of three 7-item subscales that measured depression, anxiety, and stress. The participants rated how often they had experienced these symptoms in the previous week on a 4-point scale (0 means “not at all for me,” 3 means “very well for me,” or “most of the time”), The higher the scored, the more distresses the experienced. In this study, Cronbach’s α of DASS-21 was 0.94, and that of the subscale of depression, anxiety, and stress was 0.86, 0.85, and 0.86, respectively.

### Short grit scale (grit-S)

The scale was developed by Duckworth and Quinn (2009), and Li et al. (2018) applied and validated the Chinese version. The Grit-S scale consisted of two dimensions: consistency of interest and persistence of effort. The scale contained 8-sub-items, using the Likert 5-point scale (from 1 point = “not at all like me” to 5 points = “very like me”), with higher scores indicating a higher level of grit. In this study, Cronbach’s α of the Grit scale was 0.76, and Cronbach’s α of the CI and PE subscale was 0.73 and 0.68, respectively.

### Adult dispositional hope scale (ADHS)

The scale was developed by Snyder et al. (1991), and Ren (2006) applied and validated the Chinese version. ADHS was used to assess desirability in individuals over 15 years of age. The scale consisted of 12 items, of which 4-sub-items served as fillers and were not explained. The remaining 8- projects were divided into two dimensions: path thinking and agency thinking. The ADHS scale used a 4-point scale (from 1 = “absolutely wrong” to 4 = “absolutely right”), with higher scores indicating a higher level of hope. In this study, Cronbach’s α of the hope scale was 0.89.

### Procedures

The Ethics Committee of Chengdu 4th Hospital approved this study, and the China Clinical Trials Registry registration number is CHICTR1900020715. The study was conducted by an anonymous online survey of clinical nurses. All study participants were asked to sign an informed consent form before the survey and to participate voluntarily. Each hospital had assigned a nurse to be responsible for the investigation, and researchers had trained the nurse. The survey nurse was responsible for inviting other participants to complete the anonymous online questionnaire, obtained by clicking on the web link on a mobile phone.^1^ Note that all questionnaires were self-assessed and completed by participating nurses independently. The survey nurses responded to the vague and vague items presented by participants during the field survey by the unified guidelines.

### Statistical analysis strategy

The preliminary statistical analysis was performed by SPSS 26 (IBM, Armonk, NY), including testing for correlations between the variables. Path analysis was performed using Amos 24 (IBM, Armonk, NY) to examine the mediating model relationship between hope, two components of grit (CI and PE), and three indicators of psychological distress (DAS). The hypothesis model was tested according to path coefficient and goodness of fit. Parameter estimation used maximum likelihood. Model fitting was estimated using Chi-square (χ2), degree of freedom (DF), mean square error (RMSEA), comparison fit index (CFI), normalized fit index (NFI), The goodness of fit index (GFI), corrected goodness of fit index (AGFI). The index of good fit of the model (Diamantopoulos et al., 2000) included: the fitness statistic Chi-square value was significant (*p* > 0.05), relative Chi-square (Chi/DF) < 3, RMSEA <0.08, CFI > 0.9, NFI > 0.90, GFI > 0.90, AGFI >0.90. In addition, based on 5,000 bootstrap samples, 95% confidence intervals were calculated to estimate nonstandardized coefficients for testing mediating effects.

## Results

### Descriptive statistics and relevance

Table 1 shows the mean and standard deviation of the sociodemographic variables and their relationship to the grit and hope scores. 682 clinical nurses were recruited, of whom 635 met the study criteria. The majority of nurses were female (90.4%), with a mean age of 31.61 (SD = 7.27) years, and about half of the nurses (56.9%) had a bachelor’s degree or above. Their average nursing work time was 10.51 (SD = 7.94) years.

There were no significant differences in the grit scores of nurses of different gender, ages, working years, educational levels, and positional ranks (*p* > 0.05). Nurses’ grit scores were significant differences among different types of hospitals (*p* ≤ 0.01), but for nurses in various departments except for the psychiatric department, the scores of grit were not significant differences (*p* > 0.05). In this study, nurses in psychiatric hospitals scored higher on the grit scale than in general hospitals.

There were also no significant differences in the nurses’ hope scores among different hospital types (*p* > 0.05), but among various departments, there were substantial differences (*p* = 0.002). The hope scores of nurses of different ages and working years were statistically significant (*p* ≤ 0.007), indicating that might be the higher the seniority of nurses, the higher the level of nurses’ hope. Importantly, there were also differences in the story of nurses’ hope among different positional ranks (*p* = 0.005), which means that positional ranks (career planning) might be an essential factor influencing the level of clinical nurses’ hope.

### Relationships between model variables

There was a positive correlation between the hope and both grit’s CI and PE subscales (*r* = 0.36–0.52, *p* < 0.01), and the positive correlation between the hope and PE was more potent than that of the CI. The results showed that clinical nurses with higher levels of CI and PE might also have higher levels of hope, especially PE. Both the CI and PE subscales of grit were negatively correlated with the three indicators of psychological distress (*r* = −0.21 ~ −0.38, *p* < 0.01), and the negative correlation between CI and psychological distress was more robust. It suggested that clinical nurses with higher CI and PE might have a lower risk of psychological distress. Moreover, CI seemed to be a more negative predictor of clinical nurses’ psychological distress than PE. Hope also negatively correlated with the three indicators of psychological distress (*r* = −0.33 ~ −0.37, *p* < 0.01), indicating that clinical nurses with a higher level of hope might also have a lower risk of psychological distress. The correlations between the CI, PE, hope, depression, anxiety and stress are shown in Table 2.

**Table 2:** Correlation between model variables (*n* = 635).

Varname	Mean	SD	CI	PE	Hope	Stress	Anxiety	Depression
			R (p)	R (p)	R (p)	R (p)	R (p)	R (p)
CI	12.54	2.65	1					
PE	14.6	2.35	0.39^**^	1				
Hope	21.88	3.49	0.36^**^	0.52^**^	1			
Stress	13.75	4.16	−0.38^**^	−0.21^**^	−0.35^**^	1		
Anxiety	12.3	3.99	−0.38^**^	−0.22^**^	−0.33^**^	0.83^**^	1	
Depression	11.54	3.69	−0.38^**^	−0.28^**^	−0.37^**^	0.83^**^	0.82^**^	1

** At the 0.01 level (two tailed), the correlation is significant.

### Adjustment analysis

**Figure 2:**
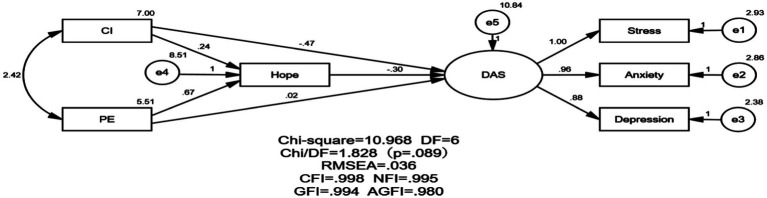
Validation model.

By using path analysis, the present work investigated the mediating role of hope in the mechanism of psychological distress influenced by CI and PE scales of grit. The goodness of fit of the model was assumed to be Χ2 = 1.828 (*p* = 0.089), DF = 6, RMSEA = 0.036, CFI = 0.998, NFI = 0.995, GFI = 0.994, AGFI = 0.980, respectively. The significance level was calculated by path estimation to verify this study’s path parameters and validity. The path loading is shown in Figure 2.

### The mediating effect of hope

The indirect effect, direct effect, and total effect of the measured path in the model indicated that hope played a part mediating role in the effect of the CI subscale of grit on the path of DAS. The direct effect (*r* = −0.47, *p* < 0.001), indirect effect (*r* = −0.07, *p* < 0.001), and total effect (*r* = −0.54, *p* < 0.001) of CI on DAS were significant, as shown in Tables 3, 4. In this work, the consistency of interest in grit directly and significantly negatively moderated psychological distress in clinical nurses, and it could also enhance the negative moderating effect on psychological distress by increasing nurses’ level of hope.

**Table 3:** Consistency of interest path analysis.

Path	Unstandardized coefficients	Standardized coefficients	S.E.	C.R.	*p*
CI → HOPE	0.24	0.18	0.05	4.99	<0.001
HOPE→DAS	−0.30	−0.28	0.05	−6.41	<0.001
CI → DAS	−0.47	−0.33	0.06	−8.10	<0.001

**Table 4:** Hope mediating effects.

Path	Utility values	SE	Bias-corrected 95% CI			Effect sizes
			Lower	Upper	*p*	
Indirect effecs	−0.07	0.02	−0.12	−0.03	<0.001	12.96%
Direct effects	−0.47	0.06	−0.59	−0.35	<0.001	87.04%
Total effects	−0.54	0.06	−0.66	−0.42	<0.001	100%

### The suppressing effect of hope

The model’s indirect effect, direct effect, and the total effect of the measured path indicated that hope obscured the effect of the PE subscale on DAS. The direct effect of PE on DAS (R = 0.02, *p* = 0.79) was not significant; The indirect effect (R = −0.2, *p* < 0.001) and the total effect (R = −0.18, *p* = 0.01) were significant (Tables 5, 6), they suggested that hope conceals the PE subscale on DAS (MacKinnon et al., 2000; MacKinnon and Lamp, 2021). In this study, hope concealed the negative moderating effect of the perseverance of effort of grit on psychological distress in clinical nurses, and the PE subscale of grit could enhance the negative moderating effect on psychological distress by raising the level of hope of nurses.

**Table 5:** Perseverance of effort path analysis.

Path	Unstandardized coefficients	Standardized coefficients	S.E.	C.R.	*p*
PE → HOPE	0.67	0.45	0.05	12.50	0.009
HOPE→DAS	−0.30	−0.28	0.05	−6.41	<0.001
PE → DAS	0.02	0.01	0.07	0.27	0.834

**Table 6:** Hope suppressing effects.

Path	Utility values	SE	Bias-corrected 95%CI			Effect sizes
			Lower	Upper	*p*	
Indirect effecs	−0.2	0.04	−0.29	−0.13	<0.001	110.63%
Direct effects	0.02	0.08	−0.14	0.17	0.834	
Total effects	−0.18	0.07	−0.31	−0.05	0.009	

## Discussion

This study aimed to explore the effects of hope and grit on psychological distress and established a path model to explain two components of grit (CI and PE) and the impact mechanism of hope on psychological distress. The results of this study are as follows.

Our research showed a low to moderate negative correlation between grit and hope with psychological distress in clinical nurses. It suggested that clinical nurses with a high level of grit or hope seem to have less psychological distress, consistent with previous findings in other populations (Berendes et al., 2010; Alacorn et al., 2013; Datu, 2021). Consistent with earlier findings in undergraduate students in the Philippines (Datu et al., 2016), We also found that the negative effect of CI on psychological distress was significantly more potent than PE. In addition, we also found a low to moderate positive correlation between hope with CI and PE, and the correlation between PE was stronger. In conclusion, grit and hope as positive traits and mental resources were negatively associated with symptoms of stress, depression, and anxiety and contributed to the mental health of clinical nurses. These findings confirmed the positive roles of grit and hope in clinical nurses’ mental health management.

The hypothetical mediation model in our study was based on the correlations between CI and PE and hope and psychological distress to test their effect mechanism. We found hope improved the negative effects of CI and PE on psychological distress to varying degrees. It revealed that increasing the level of hope of clinical nurses may more effectively reduce psychological distress. Noted that, relative to the lack of empirical research on the intervention of grit (Eskreis-Winkler, 2015; Keegan, 2017; Tang et al., 2019), many interventions of hope have been proven to be significant (Khodabakhshi et al., 2017; KhalediSardashti et al., 2018). Hope seemed to be a more critical factor for the psychological health intervention of clinical nurses because it might be of greater practical significance.

In summary, the results of this study deepen our understanding of the role of grit and hope in the psychological distress of clinical nurses. Grit and hope were the protective factors of psychological distress in clinical nurses. Nurses with higher levels of grit and hope might be less likely to experience psychological distress. The exciting fact was that hope may raise the effect of grit to reduce the risk of clinical nurses’ psychological distress. Although researchers have found several factors that can explain the psychological pain of clinical nurses (Giannetta et al., 2021; Sirois and Owens, 2021; Xu et al., 2021), hope and grit, as cultivable positive traits and psychological resources, provided a new feasible path for clinical nurses’ mental health support. They were worthy of attention in the clinical nurses’ mental health management and support.

### Limitations

The study was cross-sectional, so we could not infer cause and effect between variables. Future studies should consider longitudinal designs to investigate the relationship between grit, hope, and psychological distress. Participants in this study were clinical nurses at high risk for psychological distress, and other high-risk psychological distress populations should be considered in future studies. This study only focused on hope and grit, and future research should explore the impact of other positive psychological traits on psychological distress. Moreover, this study assessed grit, hope, and psychological distress relied on self-reports, factors that might be influenced by response bias and social desirability bias (Pan et al., 2021).

## Conclusion

This study confirmed grit and hope’s protective effect on clinical nurses’ psychological distress. The two aspects of grit (CI and PE) also significantly negatively regulate the psychological distress of clinical nurses, and hope partly mediates the effects. We also found a suppressing effect of hope in the relationship between PE and psychological distress. Our findings provide a possible reference pathway for the current prevalence of mental health support management, and the evidence obtained in the study also contributes to the development of different strategies and policies related to mental health. Furthermore, the current findings also contribute to the development of psychopathology, an emerging field at the intersection of psychology, psychiatry, and radiology (Li et al., 2021; Lai et al., 2022; Suo et al., 2022; Zhang et al., 2022).

## Data availability statement

The raw data supporting the conclusions of this article will be made available by the authors, without undue reservation.

## Ethics statement

The studies involving human participants were reviewed and approved by Committee of Chengdu 4th Hospital. The patients/participants provided their written informed consent to participate in this study.

## Author contributions

XP was involved in all aspects of the study and preparation of the manuscript. DW was involved with the design of the study and preparation of the manuscript. All authors contributed to the article and approved the submitted version.

## Funding

This work was supported by the National Natural Science Foundation of China (grant number 82001444).

## Conflict of interest

The authors declare that the research was conducted in the absence of any commercial or financial relationships that could be construed as a potential conflict of interest.

## Publisher’s note

All claims expressed in this article are solely those of the authors and do not necessarily represent those of their affiliated organizations, or those of the publisher, the editors and the reviewers. Any product that may be evaluated in this article, or claim that may be made by its manufacturer, is not guaranteed or endorsed by the publisher.

## Footnotes

^1^ http://www.wjx.CN/
